# Compact Smartphone-Based Laser Speckle Contrast Imaging Endoscope Device for Point-of-Care Blood Flow Monitoring

**DOI:** 10.3390/bios12060398

**Published:** 2022-06-09

**Authors:** Youngkyu Kim, Woo June Choi, Jungmin Oh, Jun Ki Kim

**Affiliations:** 1 Biomedical Engineering Research Center, Asan Institute for Life Science, Asan Medical Center, Seoul 05505, Korea; limexce@gmail.com (Y.K.); mini1kr@naver.com (J.O.); 2 School of Electrical and Electronics Engineering, Chung-Ang University, Seoul 06974, Korea; cecc78@cau.ac.kr; 3 Department of Convergence Medicine, College of Medicine, University of Ulsan, Seoul 05505, Korea

**Keywords:** endoscopy, point of care, smartphone, laser speckle contrast imaging, blood flow

## Abstract

Laser speckle contrast imaging (LSCI) is a powerful visualization tool for quantifying blood flow in tissues, providing simplicity of configuration, ease of use, and intuitive results. With recent advancements, smartphone and camera technologies are suitable for the development of smartphone-based LSCI applications for point-of-care (POC) diagnosis. A smartphone-based portable LSCI endoscope system was validated for POC diagnosis of vascular disorders. The endoscope consisted of compact LED and laser illumination, imaging optics, and a flexible fiberscope assembled in a 3D-printed hand-held cartridge for access to body cavities and organs. A smartphone’s rear camera was mounted thereto, enabling endoscopy, LSCI image acquisition, and processing. Blood flow imaging was calibrated in a perfused tissue phantom consisting of a microparticle solution pumped at known rates through tissue-mimicking gel and validated in a live rat model of BBN-induced bladder cancer. Raw LSCI images successfully visualized phantom flow: speckle flow index showed linearity with the pump flow rate. In the rat model, healthy and cancerous bladders were distinguishable in structure and vasculature. The smartphone-based low-cost portable mobile endoscope for monitoring blood flow and perfusion shows promise for preclinical applications and may be suitable for primary diagnosis at home or as a cost-effective POC testing assay.

## 1. Introduction

Over the years, the endoscope has proven to be an invaluable tool for primary diagnosis in modern medicine [1]. Endoscopes are routinely used in diverse medical fields, such as gastroenterology, to visualize the hollow organ interiors of the body. The device enables intuitive examination [2], minimally invasive surgery [3], and treatment [4] at different cavity sites, which cannot be achieved by other diagnostic instruments. With the growing attention to personal healthcare services, the demand for an endoscope for point-of-care testing (POCT) has increased. POCT, also known as a near-patient test, refers to medical testing performed at or near a patient’s location without sending a sample to a hospital. It provides quick, simple, and inexpensive diagnostics with acceptable accuracy in non-laboratory settings [5]. As a result, POCT technologies can improve diagnostic testing accessibility in resource-constrained areas such as remote regions or patient homes. The endoscope would be invaluable as a POCT tool. However, current clinical endoscopes are unsuitable for field diagnosis because most endoscopes require peripheral equipment to operate, such as a power supply, data-processing unit, and monitor. Portable endoscope systems combining this equipment have been developed and used to examine cavities in animals [6,7] and to evaluate the gastrointestinal tracts of human patients [8,9], which demonstrated the potential of POCT endoscopy. However, these systems are not light enough to carry (approximately 8–9 kg) and are high-priced, necessitating the development of a compact, easy-to-handle, and relatively low-cost endoscope system.

Owing to advancements in microprocessor technology, such as the central processor unit (CPU) and cutting-edge image sensors, smartphones have been equipped with more robust data processing and imaging performance [10,11,12]. For example, recent commercial smartphone models support video recording at a camera speed of over 100 frames per second (FPS) with full HD (1920 × 1080 pixels) or higher image resolution [13]. Due to such features (rapid image acquisition, high-quality display), and additional hardware improvements such as battery and data storage, smartphones have now become mobile all-in-one computers, and therefore, they have been recently utilized as preferred devices for mobile-based POCT. The smartphones have been readily combined with state-of-the-art biosensing technologies such as microfluidic chips to generate moveable diagnostic and monitoring systems for in situ medical testing [14,15,16]. Many attempts have also been made to use smartphones for POCT endoscopic imaging. Simple smartphone integration with existing endoscopes has been feasible for POCT purposes [17,18,19]. However, the use of smartphones in endoscopy is mainly confined to simple bright-field observation [18,19]. Most recently, however, a few endoscopic studies have used its high-speed data acquisition and data-processing capability for video analysis of patient vocal cords [20] or control of the articulable endoscope system [21].

Laser speckle contrast imaging (LSCI) is a reliable interferometric technique for blood flow imaging of biological tissue [22]. As a highly scattering tissue is illuminated by coherent light, laser speckles as randomized interference textures are formed on the tissue. The LSCI detects dynamic changes in the speckle signals caused by the blood flow to generate a full-field map of blood vessels in the tissue [23]. Several advantages of LSCI, such as fast vessel mapping, low cost (>90 USD), simplicity, ease of use, and high compatibility with conventional microscopes make it the preferable tool for imaging tissue perfusion [24], enabling researchers to monitor vascular changes in tissue lesions [25] or vascular responses to the tissue under stimuli [26]. Recent developments in mobile healthcare have driven smartphone use in LSCI for POCT [27,28]. Zhang et al. developed the first smartphone-based portable LSCI device and successfully utilized LSCI on the human palm [15]. All LSCI tasks (data capturing, processing, and display) were performed in real-time using an OpenGL-driven algorithm embedded in the smartphone’s graphic processing unit (GPU). However, no studies have applied the laser speckle imaging technique to mobile endoscope systems. Combined with endoscopy, LSCI can offer additional blood perfusion information and endoscopic images. This is useful for assessing internal tissue viability depending on the blood circulation [29].

In the present study, we report the development of a smartphone-based portable LSCI endoscope. To our knowledge, this is the first study to demonstrate both LSCI and endoscopy in the body’s organ cavity by using a prototype mobile endoscope system. The system performance was tested using tissue-mimicking phantom flow and a cancer model in small animals. These outcomes suggest the feasibility of endoscopic POCT.

## 2. Materials and Methods

### 2.1. Implementation of Smartphone-Based Portable LSCI Endoscope Device

**Figure 1:**
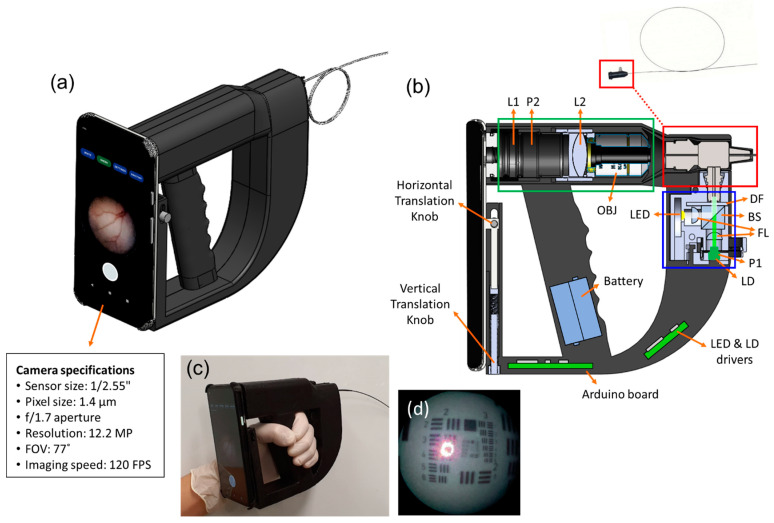
Implementation of a smartphone-based portable LSCI endoscope device: (**a**) A 3D model of a smartphone-based hand-held LSCI endoscope device and a schematic of its interior; (**b**) an image showing the light source module (blue box), fiberscope (red box), and lens unit (green box). L#: lenses, P#: linear polarizers, OBJ: objective, DF: diffuser, BS: beam splitter, FL: focusing lens, LD: laser diode. The imaging fiber bundle attached to the fiberscope (inset in (**b**)) is used as a flexible probe for endoscopy and LSCI; (**c**) a photograph of the device implemented as a prototype system (70 (width) × 150 (height) × 250 (length) mm^3^); (**d**) an endoscopy image of USAF 1951 test target.

We developed a low-cost smartphone-based portable LSCI endoscope. Figure 1a shows the 3D composition of our system, which is composed of two primary components: a smartphone and an endoscope encased in a 3D-printed handgun-shaped holder. The interior of the endoscope is shown in Figure 1b. It is composed of a light source module (blue box), fiberscope (red box), and lens unit (green box). In the light source module, a white LED (600 nm at a central peak in the spectrum, 2 W) was used for bright-field endoscopy, whereas a green laser diode (LD) (532 nm, 50 mW) was used as a coherent light source for LSCI. Both were operated using battery-powered LED and LD drivers. The drivers were controlled using an Arduino Nano Every microcontroller board (45 × 18 mm, 5 g), embedded in the system with a Bluetooth module. The lens unit was made up of two achromatic lenses (L1 and L2) and an infinity-corrected 10 × objective (OBJ). L1 (f = 50 mm) and L2 (f = 3.1 mm) were relay optics used to deliver light from the OBJ to the focusing lens on the smartphone camera. A thin fiberscope (Myriad Fiber Imaging Inc., Dudley, MA, USA) with a flexible single-mode optical fiber bundle (FIGH-30-650S, 30,000 ± 3000 pixels, 35 mm minimum bending radius, Fujikura Ltd., Tokyo, Japan) (see the inset of Figure 1b) was used as an endoscopic probe for imaging. The probe was placed between the light source module and the relay lens unit (Figure 1b). A pair of orthogonal linear polarizers (P1, P2) was inserted in front of the LD and smartphone camera to reduce strong specular reflection on the sample surface. This enabled the output beam from the light source to illuminate the interior of the body cavity via the fiberscope. The optical power of the beam irradiated at the tip of the fiberscope was 14 mW for LED and 1 mW for LD, corresponding to 0.5 mW/mm^2^ and 0.035 mW/mm^2^, respectively. Lights scattered from the interior are received by the fiberscope and transmitted to the relay lens unit. A commercial Android smartphone (Google Pixel 4a, 128 GB, 1080 × 2340 pixels, 143 g, 2020) was mounted on the endoscope using vertical and horizontal translation knobs to align the rear camera with the relay lens unit. Endoscopy images (1024 × 720 pixels) were captured using the smartphone camera at an imaging speed of 120 FPS with 10× optical magnification. 

Figure 1c shows the implemented system prototype product (70 mm (width) × 150 mm (height) × 250 mm (length), less than 1.2 kg). Real-time imaging was displayed on custom-developed smartphone software, or the captured images were saved as a video file (formatted WebM with VP8 codec) on the smartphone for post-processing. To measure image resolution, we endoscopically imaged USAF 1951 test target with our device (Figure 1d), showing that the minimum resolvable lines of the target image were 16 lp/mm corresponding to 67 μm in resolution. The image resolution was comparable to that of a commercial ureteroscope (URF-V, Olympus) [30].

Our system can provide two imaging modes for endoscopy and LSCI by using a custom-built smartphone application. In endoscopy mode, the white LED is turned on for bright-field inspection of organ tissue. In LSCI mode, the light is promptly switched to the green LD for coherent light illumination. In the LSCI mode, laser speckle patterns generated by LD illumination on the tissue were captured as raw speckle images using a smartphone camera. The speckle image contains motion information of moving scatters, such as red blood cells (RBCs), causing temporal changes in speckle signals [22]. To describe the degree of speckle fluctuation, it is normal to define the speckle contrast K as the ratio of the standard deviation of the light intensity over a region to the mean value between 0 and 1, representing the blood flow. Usually, there are two methods to map the speckle contrast K: laser speckle spatial contrast analysis (LSSCA) and laser speckle temporal contrast analysis (LSTCA). The former calculates the K with intensity values in a small window kernel of a single speckle image and the latter calculates the K with intensity values at a pixel of a time-sequence of speckle images. While LSSCA has better temporal resolution than LSTCA, it is relatively poor for spatial resolution. We used spatiotemporal laser speckle contrast analysis (STLASCA), a combined version of two methods [31,32], calculating the K with N_s_ × N_s_ × N pixel cube, where N_s_ is the length of the kernel matrix and N is the number of image frames. Therefore, STLASCA can be compromised to improve both resolutions in the speckle contrast K map [31,32]. To implement the STLASCA algorithm in this study, 15 captured raw speckle images (1024 × 720 × 15 pixels) were converted to grayscale. Then, a data cube (7 × 7 × 15 pixels) was obtained by overlaying a 7 × 7-pixel kernel on the grayscale image stack. The mean and standard deviation of the intensity of all pixels for each data cube were calculated. Their ratio generated speckle contrast K. Upon completion of the kernel sliding, the K value output formed a speckle contrast image displayed on the smartphone screen.

### 2.2. Development of a User Interface Smartphone App for Real-Time Endoscopy and LSCI

**Figure 2:**
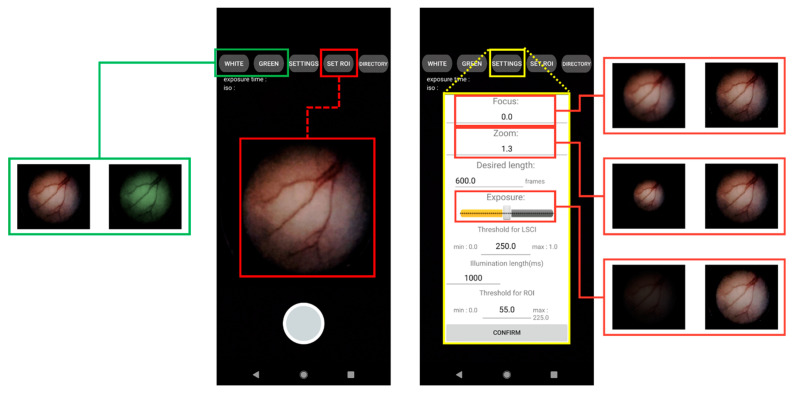
Android-based user-interface software for the smartphone LSCI endoscope device.

**Figure 3:**
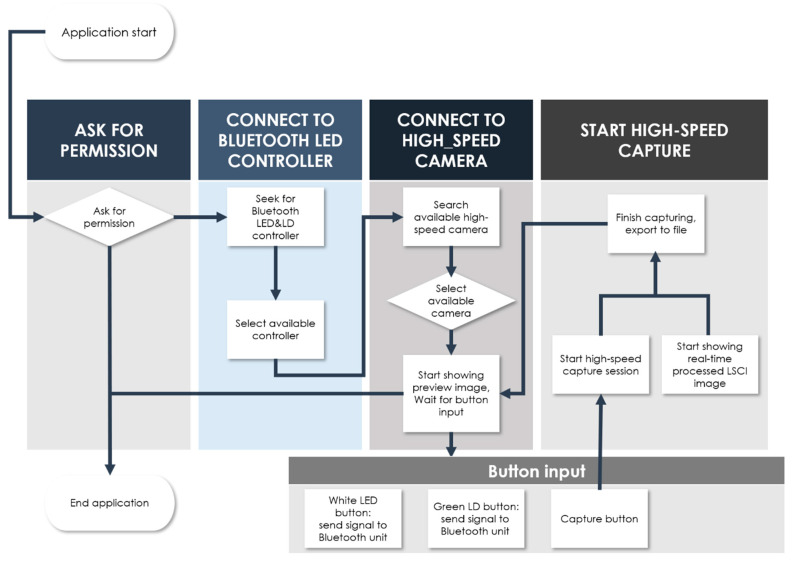
Step procedure diagram illustrating the endoscopy and LSCI operations.

We developed an Android-based smartphone software with a simple and intuitive user interface (UI), allowing users to operate endoscopy and LSCI efficiently. The software was developed with Android Studio, an official Google Android development tool, using the Java and Kotlin languages. OpenCV, an open-source image-processing library, was used for software development. Figure 2 shows the main pages of the software developed for smartphones. The figure includes button-type icons for the mobile application functions. The UI’s white and green buttons run light sources to illuminate the sample for endoscopy and LSCI, respectively. Their outputs are displayed at 120 FPS as white-light images and raw speckle images on the app screen. The “SETTINGS” button brings up a control box where users can manipulate display parameters such as focus adjustment, digital zoom, and display brightness (see right in Figure 2). The larger round button at the bottom of the app screen starts the video recording. When the recording starts, the app screen can display LSCI images (K maps) computed with the raw speckle images at video output levels of >30 FPS. The “SET ROI” button selects a region of interest (ROI) in the LSCI display. As shown in the flow chart in Figure 3, paired Bluetooth devices relay wireless communication between the app and endoscope electronics.

### 2.3. Procedure of Tissue-Mimicking Flow Phantom Manufacture

To test the flow-imaging ability of the developed device, we prepared a scattering flow phantom. To simulate an optically turbid medium, such as a tissue background, the phantom was made of polydimethylsiloxane mixed with 0.15% (15 g/100 mL) TiO_2_. A 2.5% microparticle solution was then pumped into a light-transparent 1 mm diameter silicon tube immersed in the phantom at constant flow rates ranging from 0.023 mL/min (0.5 mm/s flow speed) to 0.094 mL/min (2.0 mm/s flow speed) using a high-precision infusion syringe pump (Fusion 200, Chemyx Inc., Stafford, TX, USA). This effectively simulated the blood flow of a superficial vessel within stationary tissue. The device probe was positioned linearly 5 mm in front of the surface of the perfused phantom. The light from the probe tip illuminated the phantom for the LSCI measurement.

### 2.4. Preparation of Small Animal Model

To evaluate the feasibility of the device for functional mobile endoscopy, we conducted in vivo small animal experiments using the device. Hollow organ urinary bladders of adult Sprague Dawley rats were chosen for cystoscopy because of the ease of access through the urethra with a thin fiber probe. To investigate vascular disorders in the bladder, bladder cancer rat models were created by administering N-butyl-N-(4-hydroxybutyl) nitrosamine (BBN) to the drinking water for seven months. This BBN-treated cancer model is the most commonly used preclinical murine model of bladder carcinogenesis for accurately replicating human disease. It is responsible for high-grade invasive tumors of the urinary bladder [33,34]. The normal (*n* = 2) and bladder cancer rat models (*n* = 3) were anesthetized with an intravenous injection of 0.06% Zoletil and 0.04% Rompun per 100 g of body weight. The fully immobilized animal was placed on a temperature-controlled heating pad, and its bladder was emptied by applying gentle pressure to the bladder region. The emptied bladder was then rinsed five times with phosphate-buffered saline (PBS). Following the bladder wash, the flexible endoscope fiber tip of the device was gently inserted into the urethra and carefully directed toward the bladder site through the urinary tract. After navigating through the bladder lumen of a normal rat, the probe tip was situated at the bladder wall with vascularity. The tip was positioned at the observable tumor mass in the bladder cancer model for endoscopy and LSCI measurements.

## 3. Results

### 3.1. Tissue-Mimicking Flow Phantom Imaging

**Figure 4:**
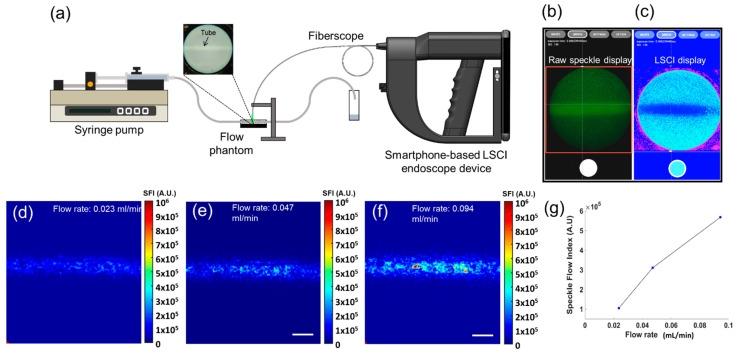
Tissue-mimicking phantom flow imaging with the smartphone-based LSCI endoscope device: (**a**) experimental setup of the phantom flow experiment; (**b**) raw speckle images of the phantom are displayed in real-time on the screen of the developed smartphone app; (**c**) laser speckle contrast images (K maps) computed with the raw speckle images displayed on the app at 30 FPS; (**d**–**f**) Speckle flow index (SFI) images of the phantom flow at different flow rates: 0.023 mL/min (**d**), 0.047 mL/min (**e**), and 0.094 mL/min (**f**); (**g**) a plot of the calculated SFIs with different flow speeds. Scale bars: 0.5 mm.

Figure 4a shows the experimental setup for tissue-mimicking phantom flow imaging using our smartphone-based LSCI endoscope device. When the “GREEN” button in the app was activated, the phantom’s green laser-induced raw speckle images were displayed (Figure 4b). They could be switched to the laser speckle contrast images in the color map (jet) display during recording (Figure 4c). In Figure 4c, the tube is clearly visible at a relatively lower contrast K. This may be due to perfusion-induced washout of the raw speckles (Figure 4b), resulting in decreased K values. To correlate the K value with the flow rate, the speckle contrast K was converted to speckle flow index (SFI), calculated as 1/(2TK^2^) [35], where T denotes the exposure time of the smartphone camera (8.33 ms in our work) during image acquisition. Figure 4d,e show the SFI images of the phantom flow at flow rates of 0.023 mL/min and 0.094 mL/min, respectively. As shown in Figure 4f, these images show a larger SFI at a higher flow speed, which is positively correlated. These results suggest that our device may be able to locate perfused vessels in turbid tissues.

### 3.2. In Vivo Small Animal Imaging

**Figure 5:**
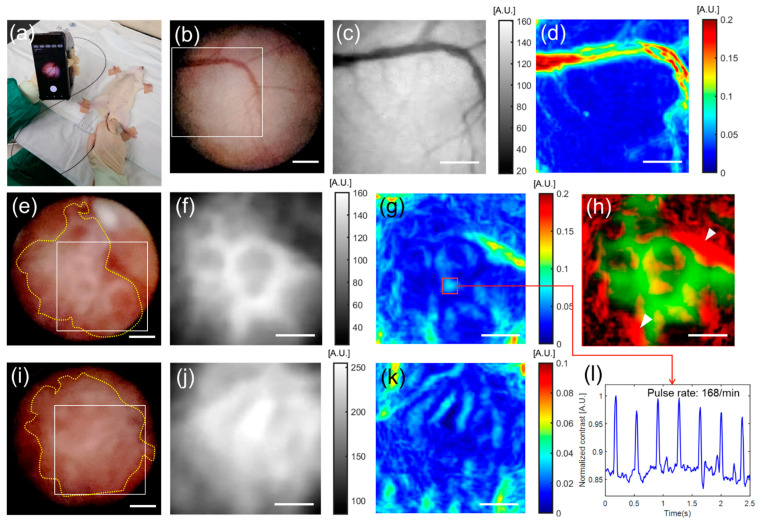
In vivo rat bladder endoscopy using the smartphone-based LSCI endoscope device: (**a**) anesthetized rat under cystoscopy operated by a clinician using the device; (**b**) white light image of the normal rat bladder, showing typically healthy bladder lumen; (**c**) grayscale raw speckle image at a region of interest (ROI) marked as a boxed area in (**b**), and corresponding laser speckle contrast image (**d**), visualizing a major bladder vessel and its branches; (**e**,**i**) white light images of the bladder cancer rat models, commonly exhibiting a mass of tumors (dotted lines) growing on the bladder wall; (**f**,**j**) grayscale speckle images of ROIs (boxed areas in (**e**,**i**)); (**g**,**k**) laser speckle contrast images of (**f**) and (**j**), delineating complex micro-vasculatures in the tumors, morphologically different from the normal vessel (**d**); (**h**) overlaid image of (**f**) (green) and (**g**) (red), depicting the tumor vascularity sprouted from major bladder vessels (arrowheads); (**l**) plot of time-course speckle contrasts recorded at a box in (**g**); the pulsatile contrast change may be due to blood pulsation caused by heartbeats. Scale bars: 1.0 mm.

After validating the flow imaging performance of the tissue-mimicking phantom flow experiment, we performed endoscopic blood flow imaging in a normal rat bladder (*n* = 2) and BBN-induced cancerous rat bladders (*n* = 3) in vivo. The results are shown in Figure 5. Figure 5a shows an endoscopy image of an anesthetized rat under cystoscopy, which was examined by a clinician using the device. The device can assist clinicians in navigating the emptied bladder cavity and observing the vasculature of the bladder interior. Endoscopic images of normal rat bladders are shown in Figure 5b–d. The white-light image (6 mm × 6 mm) in Figure 5b shows a typical healthy bladder wall structure [36]. Figure 5c shows a grayscale raw speckle image at the ROI (boxed area), as shown in Figure 5a. The corresponding laser speckle contrast image in Figure 5d shows a superficial major vessel and its branches on the bladder wall (Supplementary Video S1). Figure 5e–k show the endoscopic results of the two rat bladders with cancers. White light images (6 mm × 6 mm) in Figure 5e,i show urothelial tumors (dotted lines) growing on bladder walls [37]. It was difficult to observe the vessels embedded in the tumors in cystoscopy images. However, the laser speckle contrast images (Figure 5g,k) computed with the raw speckle images (Figure 5f,j) at the boxed areas in Figure 5e,i revealed the vasculatures in the bladder tumors. The tumor vessels were distinct from the normal bladder vessels, as shown in Figure 5d (Supplementary Video S2). Figure 5h shows a composite image of bladder tumors (green) and their vasculature (red). From these tumor angiogenesis images, we can deduce that the tumors would be fed oxygen and nutrients delivered by the tumor vascular networks connected to the surrounding bladder vessels (arrowheads). In addition, it is interesting to observe a pulsatile change in the speckle contrast in Figure 5l, obtained from the blood vessel (box in Figure 5g). This represents blood pulsation related to heartbeats. These in vivo imaging results indicate the feasibility of our device for functional field endoscopy.

## 4. Discussion

In this study, we devised and implemented a mobile endoscope system using a smartphone. We produced functional blood flow imaging using LSCI technology. Current high-end smartphones possess fast computing ability and high-quality display capabilities. This all-in-one compact computer can be effectively combined with a portable endoscope to enable the mobility of endoscopy, which is a prerequisite for POC testing.

Despite LSCI’s ability to visualize blood vessels and their blood flow, few endoscopic studies have exploited it as a real-time surgical assistance tool to aid intraoperative evaluation [25,38,39]. For example, laparoscope-incorporated LSCI has been used to visualize early changes in bowel perfusion using a rat bowel occlusion model. Furthermore, it has been used to identify the intestinal vasculature of swine during laparoscopic open surgery [38]. Current LSCI endoscope systems, however, rely on separate optical and electrical devices such as standard cameras, benchtop light sources, and personal computers with monitors [39,40]. This setup is bulky, heavy, and difficult to transport, making it unsuitable for use as a POC device. Our endoscope harnessed smartphone technology for LSCI endoscopy, simplifying the device configuration and size. It can be used as a carry-on or stand-alone medical device, which is promising for POC testing. Additionally, the use of smartphones significantly reduces the overall cost of the device, compared with the prices of conventional endoscopes and LSCI systems. Our survey of LSCI systems yielded an average price of over USD 40,000–50,000 for commercially available systems. Our device costs less than USD 1850 except for the fiberscope price—almost twenty-fold cheaper. Table 1 compares the specifications of our device with the representative commercial LSCI system [41]. Our device’s compact size and significantly lower price could make it an invaluable tool for cost-effective endoscopic POC testing.

Previous LSCI studies using smartphones have successfully demonstrated their practicality as POC testing devices. However, no studies have been published on the application of LSCI to the inner organs using a portable endoscope. In our study, we first tested the vascular imaging performance of mobile endoscopy of normal cancerous rat bladders in vivo. Disruption of blood circulation is an important indicator for evaluating the severity of vascular diseases [42,43,44,45,46,47,48]. In cancer, it is particularly well-documented that the tumor vasculature is morphologically different from the hierarchically organized normal vascular network. They can be tortuous, leaky, and disorganized [49,50]. Therefore, the unique features of the tumor vasculature compared to normal tissues allow for early cancer detection at various stages or for selective therapeutic interventions [49]. In our experiment, the LSCI of bladder cancer (Figure 5) visualized the microvessels of tumors in the bladder cavity, distinguishable from the normal bladder vessels that were barely visible in the typical white light endoscopic images. Furthermore, we could measure the blood pulses due to heartbeats in the LSCI images. Therefore, animal experiments suggest that endoscopy functionality can be highly advantageous in field surgeries and emergency treatments that require prompt identification of blood ischemia or tissue viability.

The LSCI can be possible at frame rates lower than the current camera speed (120 FPS). Yuan et al. [51] suggested that ~5 ms is a desirable exposure time of a camera for imaging blood flow in rats, and this is also achievable for the low speed (1~15 FPS) cameras by reducing their exposure times in the frame periods. However, the slow acquisition may not detect the rapid change in blood flow such as blood responses to electrical stimuli that could occur beyond the frame rates. Hence, the camera speed over at least 20 FPS may be recommendable for blood flow imaging/monitoring [52].

This study had some limitations. First, unlike conventional clinical LSCI using near-infrared lasers operating at 700–800 nm, we used green lasers operating at 523 nm as the LSCI light source. The visible image sensor of the standard consumer smartphone camera was considered because of its relatively high spectral responsivity of 500–550 nm wavelength [53]. However, we observed strong light absorption at the superficial large bladder wall blood vessels, resulting in lower detection of scattered light signals from these vessels. We adjusted for this by reducing the light intensity, which may unintentionally elevate the speckle contrast K. For higher-fidelity LSCI measurements, therefore, the green laser light source could be replaced with a red light source (~660 nm) in the proposed device. This can nominally increase the speckle intensity more than 140 times.

Second, the maneuvering of the endoscope probe while viewing the smartphone screen may compel divergence between the site of the manipulating hand and the clinician’s field of view. This may be due to impaired eye-hand coordination, which is a critical issue in routine endoscopic procedures [54]. To mitigate poor hand–eye coordination, an augmented reality (AR) display may be used for endoscopic procedures [55,56,57]. AR is an interactive interface that provides a computer-generated visual overlay on real-time images. For our device, a wearable AR display, such as smart glass or a head-mounted display (HMD), would allow the clinician to look at the duplicated smartphone screen and their hands at the same time, matching the line of sight between the endoscope and the clinician.

## 5. Conclusions

The research team successfully built a novel smartphone-based real-time LSCI endoscope. This portable and compact medical device offers both endoscopy and blood flow monitoring in the body. The system was validated by tissue-mimicking phantom blood flow and live animal tests. We expect this prototype system to become an effective alternative for field and POC endoscopy.

## Figures and Tables

**Table 1:** Specification comparison of the prototype smartphone-based LSCI endoscope device and a representative commercial LSCI system [39].

Specification	Our Smartphone-Based LSCI System		Commercial LSCI System
Price	3D-printed case: USD 300	Total: Less than USD 1850.	More than USD 40,000
	LD + LED light sources: USD 200		
	Optical components: USD 1000		
	Pixel 4a smartphone: USD 350		
Camera bit depth	8 bits		12 bits
Wavelength	532 nm		785 nm
Display resolution	400 × 400 pixels		656 × 494 pixels
Data acquisition speed	120 FPS		Up to 120 FPS
Dimensions	70 × 150 × 250 mm^3^		500 × 500 × 300 mm^3^ (PC not included)

## Data Availability

Original and raw data files are available from the authors upon reasonable request.

## Supplementary Materials

The following are available online at https://www.mdpi.com/article/10.3390/bios12060398/s1, Supplementary Video S1: LSCI mapping showing normal bladder vessel, Supplementary Video S2: LSCI mapping video showing tumor vessel in BBN model, Supplementary Code: Snippet code for android application.

## References

[1] Gulati S., Patel M., Emmanuel A., Haji A., Hayee B., Neumann H. (2020). The Future of Endoscopy: Advances in Endoscopic Image Innovations. Dig. Endosc..

[2] Yamada M., Saito Y., Imaoka H., Saiko M., Yamada S., Kondo H., Takamaru H., Sakamoto T., Sese J., Kuchiba A. (2019). Development of a Real-Time Endoscopic Image Diagnosis Support System Using Deep Learning Technology in Colonoscopy. Sci. Rep..

[3] de Moura D.T.H., Aihara H., Thompson C.C. (2019). Robotic-Assisted Surgical Endoscopy: A New Era for Endoluminal Therapies. VideoGIE.

[4] Cappell M.S. (2010). Therapeutic Endoscopy for Acute Upper Gastrointestinal Bleeding. Nat. Rev. Gastroenterol. Hepatol..

[5] Nayak S., Blumenfeld N.R., Laksanasopin T., Sia S.K. (2017). Point-of-Care Diagnostics: Recent Developments in a Connected Age. Anal. Chem..

[6] Murray M.J. (2010). Endoscopy in Sharks. Vet. Clin. N. Am. Exot. Anim. Pract..

[7] Divers S.J., Boone S.S., Hoover J.J., Boysen K.A., Killgore K.J., Murphy C.E., George S.G., Camus A.C. (2009). Field Endoscopy for Identifying Gender, Reproductive Stage and Gonadal Anomalies in Free-Ranging Sturgeon (*Scaphirhynchus*) from the Lower Mississippi River. J. Appl. Ichthyol..

[8] Kang D., Lim C.-H., Choi M.-G., Lee H., Kim J.S., Cho Y.K., Park J.M., Cho Y.S., Lee B.I., Lee I.S. (2019). An Operable, Portable, and Disposable Ultrathin Endoscope for Evaluation of the Upper Gastrointestinal Tract. Dig. Dis. Sci..

[9] Choi J.H. (2014). Comparison of a Novel Bedside Portable Endoscopy Device with Nasogastric Aspiration for Identifying Upper Gastrointestinal Bleeding. WJG.

[10] McCracken K.E., Yoon J.-Y. (2016). Recent Approaches for Optical Smartphone Sensing in Resource-Limited Settings: A Brief Review. Anal. Methods.

[11] Agu E., Pedersen P., Strong D., Tulu B., He Q., Wang L., Li Y. The Smartphone as a Medical Device: Assessing Enablers, Benefits and Challenges. Proceedings of the 2013 IEEE International Workshop of Internet-of-Things Networking and Control (IoT-NC).

[12] Zhang D., Liu Q. (2016). Biosensors and Bioelectronics on Smartphone for Portable Biochemical Detection. Biosens. Bioelectron..

[13] Griffiths A.D., Herrnsdorf J., Strain M.J., Dawson M.D. (2019). Scalable Visible Light Communications with a Micro-LED Array Projector and High-Speed Smartphone Camera. Opt. Express.

[14] Liu J., Geng Z., Fan Z., Liu J., Chen H. (2019). Point-of-Care Testing Based on Smartphone: The Current State-of-the-Art (2017–2018). Biosens. Bioelectron..

[15] Geng Z., Zhang X., Fan Z., Lv X., Su Y., Chen H. (2017). Recent Progress in Optical Biosensors Based on Smartphone Platforms. Sensors.

[16] Sivakumar R., Lee N.Y. (2021). Recent Progress in Smartphone-Based Techniques for Food Safety and the Detection of Heavy Metal Ions in Environmental Water. Chemosphere.

[17] Bae J.K., Vavilin A., You J.S., Kim H., Ryu S.Y., Jang J.H., Jung W. (2017). Smartphone-Based Endoscope System for Advanced Point-of-Care Diagnostics: Feasibility Study. JMIR Mhealth Uhealth.

[18] Çelikoyar M.M., Aktas O.T. (2018). Endoscopy in Otolaryngology Utilising Smartphone as the Capturing Device. J. Vis. Commun. Med..

[19] Ha J.H.I., Sagili S.R. (2019). Smartphone Adaptor Use for Nasal Endoscopy. Eye.

[20] Kim Y., Oh J., Choi S.-H., Jung A., Lee J.-G., Lee Y.S., Kim J.K. (2021). A Portable Smartphone-Based Laryngoscope System for High-Speed Vocal Cord Imaging of Patients with Throat Disorders: Instrument Validation Study. JMIR Mhealth Uhealth.

[21] Moon Y., Oh J., Hyun J., Kim Y., Choi J., Namgoong J., Kim J.K. (2020). Cost-Effective Smartphone-Based Articulable Endoscope Systems for Developing Countries: Instrument Validation Study. JMIR Mhealth Uhealth.

[22] Boas D.A., Dunn A.K. (2010). Laser Speckle Contrast Imaging in Biomedical Optics. J. Biomed. Opt..

[23] Briers D., Duncan D.D., Hirst E., Kirkpatrick S.J., Larsson M., Steenbergen W., Stromberg T., Thompson O.B. (2013). Laser Speckle Contrast Imaging: Theoretical and Practical Limitations. J. Biomed. Opt.

[24] Heeman W., Steenbergen W., van Dam G.M., Boerma E.C. (2019). Clinical Applications of Laser Speckle Contrast Imaging: A Review. J. Biomed. Opt..

[25] Armitage G.A., Todd K.G., Shuaib A., Winship I.R. (2010). Laser Speckle Contrast Imaging of Collateral Blood Flow during Acute Ischemic Stroke. J. Cereb. Blood Flow. Metab..

[26] Li D.-Y., Xia Q., Yu T.-T., Zhu J.-T., Zhu D. (2021). Transmissive-Detected Laser Speckle Contrast Imaging for Blood Flow Monitoring in Thick Tissue: From Monte Carlo Simulation to Experimental Demonstration. Light Sci. Appl..

[27] Kong P., Xu H., Li R., Huang G., Liu W. (2021). Laser Speckle Contrast Imaging Based on a Mobile Phone Camera. IEEE Access.

[28] Jakovels D., Saknite I., Krievina G., Zaharans J., Spigulis J. Mobile Phone Based Laser Speckle Contrast Imager for Assessment of Skin Blood Flow. Proceedings of the Eighth International Conference on Advanced Optical Materials and Devices.

[29] Guven G., Hilty M.P., Ince C. (2020). Microcirculation: Physiology, Pathophysiology, and Clinical Application. Blood Purif..

[30] Zilberman D.E., Lipkin M.E., Ferrandino M.N., Simmons W.N., Mancini J.G., Raymundo M.E., Zhong P., Preminger G.M. (2011). The Digital Flexible Ureteroscope: In Vitro Assessment of Optical Characteristics. J. Endourol..

[31] Duncan D.D., Kirkpatrick S.J. Spatio-temporal algorithms for processing laser speckle imaging data. Proceedings of the SPIE BiOS 2008.

[32] Qiu J. (2010). Spatiotemporal Laser Speckle Contrast Analysis for Blood Flow Imaging with Maximized Speckle Contrast. J. Biomed. Opt..

[33] Degoricija M., Korac-Prlic J., Vilovic K., Ivanisevic T., Haupt B., Palada V., Petkovic M., Karaman I., Terzic J. (2019). The Dynamics of the Inflammatory Response during BBN-Induced Bladder Carcinogenesis in Mice. J. Transl. Med..

[34] Vasconcelos-Nóbrega C., Colaço A., Lopes C., Oliveira P.A. (2012). Review: BBN as an Urothelial Carcinogen. In Vivo.

[35] White S.M., Valdebran M., Kelly K.M., Choi B. (2018). Simultaneous Blood Flow Measurement and Dermoscopy of Skin Lesions Using Dual-Mode Dermascope. Sci. Rep..

[36] Zlatev D.V., Altobelli E., Liao J.C. (2015). Advances in Imaging Technologies in the Evaluation of High-Grade Bladder Cancer. Urol. Clin. N. Am..

[37] Yamamoto S., Fukuhara H., Karashima T., Inoue K. (2020). Real-World Experience with 5-Aminolevulinic Acid for the Photodynamic Diagnosis of Bladder Cancer: Diagnostic Accuracy and Safety. Photodiagnosis Photodyn. Ther..

[38] Zheng C., Lau L.W., Cha J. (2018). Dual-Display Laparoscopic Laser Speckle Contrast Imaging for Real-Time Surgical Assistance. Biomed. Opt. Express.

[39] Heeman W., Dijkstra K., Hoff C., Koopal S., Pierie J.-P., Bouma H., Boerma E.C. (2019). Application of Laser Speckle Contrast Imaging in Laparoscopic Surgery. Biomed. Opt. Express.

[40] Potapova E.V., Seryogina E.S., Dremin V.V., Stavtsev D.D., Kozlov I.O., Zherebtsov E.A., Mamoshin A.V., Ivanov Y.V., Dunaev A.V. (2020). Laser Speckle Contrast Imaging of Blood Microcirculation in Pancreatic Tissues during Laparoscopic Interventions. Quantum Electron..

[41] RFLSI III Laser Speckle Imaging System Flyer V1.0 2021. https://www.rwdstco.com/product-item/laser-speckle-imaging-system.

[42] Cousins C.C., Chou J.C., Greenstein S.H., Brauner S.C., Shen L.Q., Turalba A.V., Houlihan P., Ritch R., Wiggs J.L., Knepper P.A. (2019). Resting Nailfold Capillary Blood Flow in Primary Open-Angle Glaucoma. Br. J. Ophthalmol..

[43] Chang H.-Y., Yazdani A., Li X., Douglas K.A.A., Mantzoros C.S., Karniadakis G.E. (2018). Quantifying Platelet Margination in Diabetic Blood Flow. Biophys. J..

[44] Chen W., Deng Y., Jiang H., Wang J., Zhong J., Li S., Peng L., Wang B., Yang R., Zhang H. (2018). Microvascular Abnormalities in Dry Eye Patients. Microvasc. Res..

[45] Kallinowski F., Vaupel P. (1988). PH Distributions in Spontaneous and Isotransplanted Rat Tumours. Br. J. Cancer..

[46] Lip G.Y., Chin B.S., Blann A.D. (2002). Cancer and the Prothrombotic State. Lancet Oncol..

[47] Vaupel P., Kallinowski F., Okunieff P. (1989). Blood Flow, Oxygen and Nutrient Supply, and Metabolic Microenvironment of Human Tumors: A Review. Cancer Res..

[48] Jain R.K., Ward-Hartley K. (1984). Tumor Blood Flow-Characterization, Modifications, and Role in Hyperthermia. IEEE Trans. Son. Ultrason..

[49] Ruoslahti E. (2002). Specialization of Tumour Vasculature. Nat. Rev. Cancer.

[50] Siemann D.W. (2011). The Unique Characteristics of Tumor Vasculature and Preclinical Evidence for Its Selective Disruption by Tumor-Vascular Disrupting Agents. Cancer Treat. Rev..

[51] Yuan S., Devor A., Boas D.A., Dunn A.K. (2005). Determination of optimal exposure time for imaging of blood flow changes with laser speckle contrast imaging. Appl. Opt..

[52] Alex O. (2009). Holcombe, Seeing slow and seeing fast: Two limits on perception. Trends Cogn. Sci..

[53] Burggraaff O., Schmidt N., Zamorano J., Pauly K., Pascual S., Tapia C., Spyrakos E., Snik F. (2019). Standardized Spectral and Radiometric Calibration of Consumer Cameras. Opt. Express.

[54] Wentink B. (2001). Eye-Hand Coordination in Laparoscopy—An Overview of Experiments and Supporting Aids. Minim. Invasive Ther. Allied Technol..

[55] Koesveld J.J.M., Tetteroo G.W.M., Graaf E.J.R. (2003). Use of Head-Mounted Display in Transanal Endoscopic Microsurgery. Surg. Endosc..

[56] Ishioka J., Kihara K., Higuchi S., Nakayama T., Takeshita H., Yoshida S., Nakanishi Y., Kijima T., Matsuoka Y., Numao N. (2014). New Head-Mounted Display System Applied to Endoscopic Management of Upper Urinary Tract Carcinomas. Int. Braz. J. Urol..

[57] van Lindert E.J., Grotenhuis J.A., Beems T. (2004). The Use of a Head-Mounted Display for Visualization in Neuroendoscopy. Comput. Aided Surg..

