# Supplementary material for: Compact Smartphone-Based Laser Speckle Contrast Imaging Endoscope Device for Point-of-Care Blood Flow Monitoring
Source: Biosensors (Basel). 2022 Jun 9;12(6):398. doi: 10.3390/bios12060398 (PMC9220785; doi:10.3390/bios12060398)
Supplement: Supplementary file 1 [file biosensors-12-00398-s001.zip › Supplementary Code.pdf]

```
Following code snippet shows the Bluetooth device connection.


fun refresh() {
    listItem.clear()
    if (btAdapter != null) {
        for (device in btAdapter!!.bondedDev)
            if (device.getType() != BluetoothDevice.DEVICE_TYPE_LE)
            {listItem.add(device)}
    }
    listItem.sortWith(Comparator { a: BluetoothDevice, b: BluetoothDevice -
> compareTo(a, b) })
    listAdapter?.notifyDataSetChanged()

}//refresh and show list of available BT device in list UI.

override fun onListItemClick(l: ListView, v: View, position: Int, id: Long)
{
    val device = listItem[position - 1]
    val btargs = Bundle()
    btargs.putString("device", device.address)
…

    fragment.arguments = btargs

….

    Navigation.findNavController(requireActivity(),
R.id.fragment_container)
        .navigate(
            DevicesFragmentDirections.actionBluetoothToSelector(
                device.address
            ),
         )
//put the bt device in arguments bundle and transact the information into
next fragment.

}


The next code snippet shows
User open up an thread to process the image data on recording surface while
recording the original high-speed video.



val displayingthread =
Thread
{
while (isrunning)
         {

         PixelCopy.request(fragmentCameraBinding.viewFinder.holder.surface,
//surface holding high-speed visual data
                             screentobitmap,
                             PixelCopy.OnPixelCopyFinishedListener { },
                             it.handler)

           Utils.bitmapToMat(screentobitmap, matrx)
 // Convert to grayscale with OpenCV library
```

```
          Core.bitwise_or(matrx,rectangleforbgmask,cropfg)
          Core.split(cropfg,channels)
//extract only green channel of the image with OpenCV library

          channels[1].convertTo(vmatrx, CV_32FC1)
          when(counter){
               1 -> {
                     processmtrx[1] = vmatrx.clone()
                     }
               2 -> {
                     processmtrx[2] = vmatrx.clone()

                                             ...
               maxnumber ->{
                     processmtrx[maxnumber] = vmatrx.clone()
                     counter = 0
                      canbeprocessed = true
//uses Mat.clone() method instead of = calculator. If = calculator is used
the copied variable shares same memory with the original, therefore
rendering any further process on the variable worthless unless it is done
in the original.
                     }
            }
            counter++
           if(canbeprocessed){
// start processing the image only the subsequent amount of frame data
provided
                          Core.divide(processmtrx[1],scalardivision,proces
                          sdivmtrx[1])
                          Core.divide(processmtrx[2],scalardivision,proces
                          ssdivmtrx[2])
                                             …
                          Core.divide(processmtrx[maxnumber],scalardivisio
                          n,processdivmtrx[maxnumber])
//scalardivision = 1/maxnumber. Other variable is used for the division
because OpenCV only accepts scalar values. Also, division is done first
because Mat data format of Opencv has maximum value, therefore adding up
the Mat would max out therefore resulting in data loss.

                          summtrx = processdivmtrx[1].clone()
                          Imgproc.accumulate(processdivmtrx[2],summtrx)

                                       ….

                          Imgproc.accumulate(processdivmtrx[maxnumber],sum
                                      mtrx)

                          meanmatrx = summtrx.clone()


//finished deriving mean matrix needed for the LSCI intensity calculation
                          Core.absdiff(processmtrx[1],meanmatrx,processsqm
                          trx[1])
                                             ….
```

```
Core.absdiff(processmtrx[maxnumber],meanmatrx,processsqmtrx[maxnumber])
//getting absolute difference between average and the original for the standard deviation calculation
Core.multiply(processsqmtrx[1],processsqmtrx[1],processsqermtrx[1])
…
Core.multiply(processsqmtrx[maxnumber],processsqmtrx[maxnumber],processsqermtrx[maxnumber])
//getting square of absolute difference between average and the original for the standard deviation calculation
Core.divide(processsqermtrx[1],scalardivision,valmtrx[1])
……
Core.divide(processsqermtrx[maxnumber],scalardivision,valmtrx[maxnumber])
valsummtrx=valmtrx[1].clone()
Imgproc.accumulate(valmtrx[2],valsummtrx)
……
Imgproc.accumulate(valmtrx[maxnumber],valsummtrx)
//finished deriving the average of the standard deviation

Core.sqrt(valsummtrx ,matrxfinresult)
Core.divide(matrxfinresult,meanmatrx,calcmtrx)
//finished deriving the average of the standard deviation
Imgproc.threshold(calcmtrx,matrxresult,saturationlimit ,1.0, THRESH_TRUNC)
saturationmax = minMaxLoc(calcmtrx).maxVal
matrxresult.convertTo(matrxresult,CV_32FC1,1/saturationlimit)
Core.multiply(matrxresult,scalardivision2,threshmtrx)
```

```
                }
        else{

                                    ……

        }

dispmtrx = Mat(matrxresult.size(), CV_8UC1)
threshmtrx.convertTo(dispmtrx, CV_8UC1)
threshmtrx.convertTo(matfoctolo, CV_8UC3)
Imgproc.applyColorMap(dispmtrx,matfoctolo, COLORMAP_RAINBOW)
Utils.matToBitmap(matfoctolo, displayedbitmap)
handler.sendMessage(handler.obtainMessage())

//threshold, normalize and map it into rainbow color scale


                                    ….

}
displayingthread.start()
```

The following is arduino Bluetooth receiving part.

```
#include <SoftwareSerial.h>

int blueTx=2;   //Tx
int blueRx=3;   //Rx
int R_Pin=4;   //RED
int G_Pin=5;   //GREEN
int B_Pin=6;   //GREEN
char Recieve_data;
SoftwareSerial BTSerial(blueTx, blueRx);

void setup() {
  BTSerial.begin(9600);
  pinMode(LED_Pin, OUTPUT);
  pinMode(LD_Pin, OUTPUT);
  digitalWrite(LED_Pin, LOW);
  digitalWrite(LD_Pin, LOW);
  BTSerial.write("\n");

}

void loop() {

  if (BTSerial.available())
  {
    Recieve_data=BTSerial.read();
  }

  switch (Recieve_data)
  {
    case  'a':
```

```
    digitalWrite(LED_Pin,HIGH);
    BTSerial.write(BTSerial.read());
    BTSerial.write("\n");
    Recieve_data=0;
    break;

   case  'b':
    digitalWrite(LED_Pin,LOW);
    BTSerial.write(BTSerial.read());
    BTSerial.write("\n");
    Recieve_data=0;
    break;

   case  'c':
    digitalWrite(LD_Pin,HIGH);
    BTSerial.write(BTSerial.read());
    BTSerial.write("\n");
    Recieve_data=0;
    break;

   case  'd':
    digitalWrite(LD_Pin,LOW);
    BTSerial.write(BTSerial.read());
    BTSerial.write("\n");
    Recieve_data=0;
    break;
   default:
    // if nothing else matches, do the default
    // default is optional
    break;

  }


}
```
